# A novel nine-microRNA-based model to improve prognosis prediction of renal cell carcinoma

**DOI:** 10.1186/s12885-022-09322-9

**Published:** 2022-03-12

**Authors:** Chen Xu, Hui Zeng, Junli Fan, Wenjie Huang, Xiaosi Yu, Shiqi Li, Fubing Wang, Xinghua Long

**Affiliations:** 1 Department of Laboratory Medicine, Zhongnan Hospital of Wuhan University, Wuhan, China; 2 Center of Clinical Laboratory, Hangzhou Ninth People’s Hospital, Hangzhou, China; 3 Center for Single-Cell Omics and Tumor Liquid Biopsy, Zhongnan Hospital of Wuhan University, Wuhan, China

**Keywords:** Renal cell carcinoma, miRNA, Prognosis, Immune microenvironment, Immunotherapy

## Abstract

**Background:**

With the improved knowledge of disease biology and the introduction of immune checkpoints, there has been significant progress in treating renal cell carcinoma (RCC) patients. Individual treatment will differ according to risk stratification. As the clinical course varies in RCC, it has developed different predictive models for assessing patient’s individual risk. However, among other prognostic scores, no transparent preference model was given. MicroRNA as a putative marker shown to have prognostic relevance in RCC, molecular analysis may provide an innovative benefit in the prophetic prediction and individual risk assessment. Therefore, this study aimed to establish a prognostic-related microRNA risk score model of RCC and further explore the relationship between the model and the immune microenvironment, immune infiltration, and immune checkpoints. This practical model has the potential to guide individualized surveillance protocols, patient counseling, and individualized treatment decision for RCC patients and facilitate to find more immunotherapy targets.

**Methods:**

Downloaded data of RCC from the TCGA database for difference analysis and divided it into a training set and validation set. Then the prognostic genes were screened out by Cox and Lasso regression analysis. Multivariate Cox regression analysis was used to establish a predictive model that divided patients into high-risk and low-risk groups. The ENCORI online website and the results of the RCC difference analysis were used to search for hub genes of miRNA. Estimate package and TIMER database were used to evaluate the relationship between risk score and tumor immune microenvironment (TME) and immune infiltration. Based on Kaplan-Meier survival analysis, search for immune checkpoints related to the prognosis of RCC.

**Results:**

There were nine miRNAs in the established model, with a concordance index of 0.702 and an area under the ROC curve of 0.701. Nine miRNAs were strongly correlated with the prognosis (*P* < 0.01), and those with high expression levels had a poor prognosis. We found a common target gene PDGFRA of hsa-miR-6718, hsa-miR-1269b and hsa-miR-374c, and five genes related to ICGs (KIR2DL3, TNFRSF4, LAG3, CD70 and TNFRSF9). The immune/stromal score, immune infiltration, and immune checkpoint genes of RCC were closely related to its prognosis and were positively associated with a risk score.

**Conclusions:**

The established nine-miRNAs prognostic model has the potential to facilitate prognostic prediction. Moreover, this model was closely related to the immune microenvironment, immune infiltration, and immune checkpoint genes of RCC.

**Supplementary Information:**

The online version contains supplementary material available at 10.1186/s12885-022-09322-9.

## Introduction

In the latest update of GLOBOCAN worldwide cancer statistics, it was estimated that approximately 431,288 new cases of kidney cancer were diagnosed in 2020, with about 179,368 patients succumbing to the disease (https://gco.iarc.fr/today/data/factsheets/cancers/29-Kidney-fact-sheet.). Renal cell carcinoma (RCC) accounts for 90% of kidney cancers. RCC can be broken down into three major histological subtypes: clear cell RCC (CCRCC), papillary RCC (PRCC), and chromophobe RCC (ChRCC) [1–3] . These three types of RCC account for 65–70%, 15–20%, and 5–7%, respectively. Patients with CCRCC have the worst disease-specific survival because it is discovered at a more advanced stage [4]. Considerable progress has been made in treating patients with RCC, with innovative immunotherapy making dramatic advances in the management of this disease [5]. At present, the main treatment methods for RCC are: first-line combined treatment of metastatic RCC combined with immune checkpoint blocking, combining immune checkpoint blockade with tyrosine kinase inhibitors, and combination therapy with anti–vascular endothelial growth factor antibodies [6]. Immune checkpoints (IC) are inhibitory regulators of the immune system that are crucial for maintaining self-tolerance. The IC pathways are mechanisms adopted by cancer cells to disguise themselves as regular components of the human body to undergo immune evasion [7]. At the same time, immune infiltration of tumors is closely associated with clinical outcomes in RCC [8, 9].

MicroRNAs (miRNAs) can regulate various target genes and are thus involved in the regulation of various of physiological and pathological processes, including cell proliferation, fundamental biological processes, signal pathways, tumor formation and development and so on [10–13]. According to their effects on tumor development, miRNAs are classified as carcinogenic or tumor-suppressive. As an essential determinant of post-transcriptional regulation, miRNA participates in the occurrence and progression of cancer and regulates anti-cancer immune response [14].

In this study, we are committed to finding miRNAs related to the prognosis of RCC and constructing a prognostic model through lasso and multivariate cox regression, further looking for the relationship between the prognostic model and immune infiltration/ checkpoints. This research results should contribute to the immunotherapy and prognosis of RCC.

## Materials and methods

### Data preparation

Gene expression profiles and miRNA expression profiles of RCC samples were downloaded from the TCGA data portal (https://tcga-data.nci.nih.gov/tcga/, April 2021). Clinical data of RCC samples were downloaded from the website (https://www.cbioportal.org/). After excluding samples with incomplete information, 865 samples were included in the study, including 512 KIRC, 288 KIRP and 65 KICH. A list of 79 immune checkpoint genes (ICGs) was obtained from the literature (PMID: 32814346) [15]. The downloaded mRNA and miRNA raw data were integrated and standardized using the Edger software package, and differences were analyzed to obtain differentially expressed genes and their expression level. The data provided by TCGA are public and open-ended, and therefore does not require the approval of a local ethics committee.

### Construction of prognostic model for RCC

Based on the results of the difference analysis, univariate cox analysis was carried out using the R (vision 4.0.4) package ‘survival’. L1-penalized (lasso) characterized by simultaneous variable selection and shrinkage is a valuable method for determining interpretable prediction rules in high-dimensional data [16]. Based on the ‘glmnet’ package in R, lasso cox regression analysis was applied to build an optimal prognostic signature for RCC samples. The optimal values of the penalty parameter λ were determined through 26 cross-validations. Finally, multivariate cox analyses were conducted to compare their predictive power of prognostic. An equation for calculating risk score was generated based on the expression levels of these prognostic miRNAs and their regression coefficients from the multivariate cox analyses as follows:

Risk score = coefmiRNA1 x exprmiRNA1 + coefmiRNA2 x exprmiRNA2+ · ···· + coefmiRNAn x exprmiRNAn.

According to the median, the RS values for each sample were calculated, and the samples were divided into high-risk and low-risk groups. The forest plot was drawn by ‘survminer’ in R language.

### Survival analysis of prognostic model

Overall survival (OS) time and recurrence-free survival (RFS) time were used as variables to calculate the scores of 3 years and 5 years. Nomogram and calibration charts were drawn using ‘rms’ package in R language. The risk groups’ discrepancies in OS time and RFS time were analyzed using Kaplan-Meier (KM) survival analysis and the log-rank test. Survival analysis uses GraphPad Prism 8.4.2 analysis, and *p*-value < 0.05 was considered significant. ROC curve was used to estimate the prediction performance of the prognosis index and carried out using the R package ‘timeROC’.

### Subgroup analysis of clinicopathological factors

The TNM stage, age, gender and other indicators of the patients were included in the study to analyze the influence of these factors on the prognosis of the patients by log-rank-test. And univariate and multivariate cox analyses were conducted to compare their predictive power of prognostic. These analyses used the R language and GraphPad Prism, and *p*-value < 0.05 was considered significant.

### Construction of miRNA-mRNA regulatory network

To further study the interactions between miRNAs and hub genes, the Encyclopedia of RNA Interactomes (ENCORI)(http://starbase.sysu.edu.cn/; version 3.0) was utilized to predict the targeted miRNAs of hub genes. In our study, the targeted miRNAs of hub genes were defined according to the positive results of ≥3 miRNA-target predicting databases, including PicTar, TargetScan, PITA, and miRanda. After taking the intersection with the hub genes selected by the difference analysis in the training set, the Venn diagram was drawn on the Venny online website (Venny 2.1.0 (csic.es)). Finally, the interaction network of miRNAs and hub genes was constructed using Cytoscape.

### Correlation between risk score and immune infiltration

TIMER includes 10,897 samples across 32 cancer types from TCGA to estimate the abundance of six TIIC subsets (B cells, CD4 T cells, CD8 T cells, macrophages, neutrophils, and dendritic cells) [17]. The information of the six immune cells of RCC was downloaded from the TIMER website (http://cistrome.dfci.harvard.edu/TIMER/). At the same time, the stromal/ immune score of RCC was downloaded from the ESTIMATE website (https://bioinformatics.mdanderson.org/estimate/). The Wilcoxon test was used to compare the immune infiltration differences between high and low-risk groups. The log-rank test was used to analyze the relationship between stromal/immune score and OS of RCC. Finally, we analyzed the correlation between the risk score and the six immune cells.

### Correlation between risk score and immune checkpoint genes

Immune checkpoint genes (ICGs) play critical roles in circumventing self-reactivity and represent a novel target to develop treatments for cancers [18]. Using the ‘vlookup’ function in Excel, 76 immune checkpoint genes obtained from the literature were matched with the gene expression profiles of RCC. Differential analysis was done using the Edger package with *p*-value < 0.05 and HR > 1. The log-rank test was used to analyze the relationship between ICGs and OS of RCC, and the Wilcoxon test was used to compare the ICGs between high and low-risk groups.

## Results

### Difference analysis and patients grouping

**Fig. 1:**
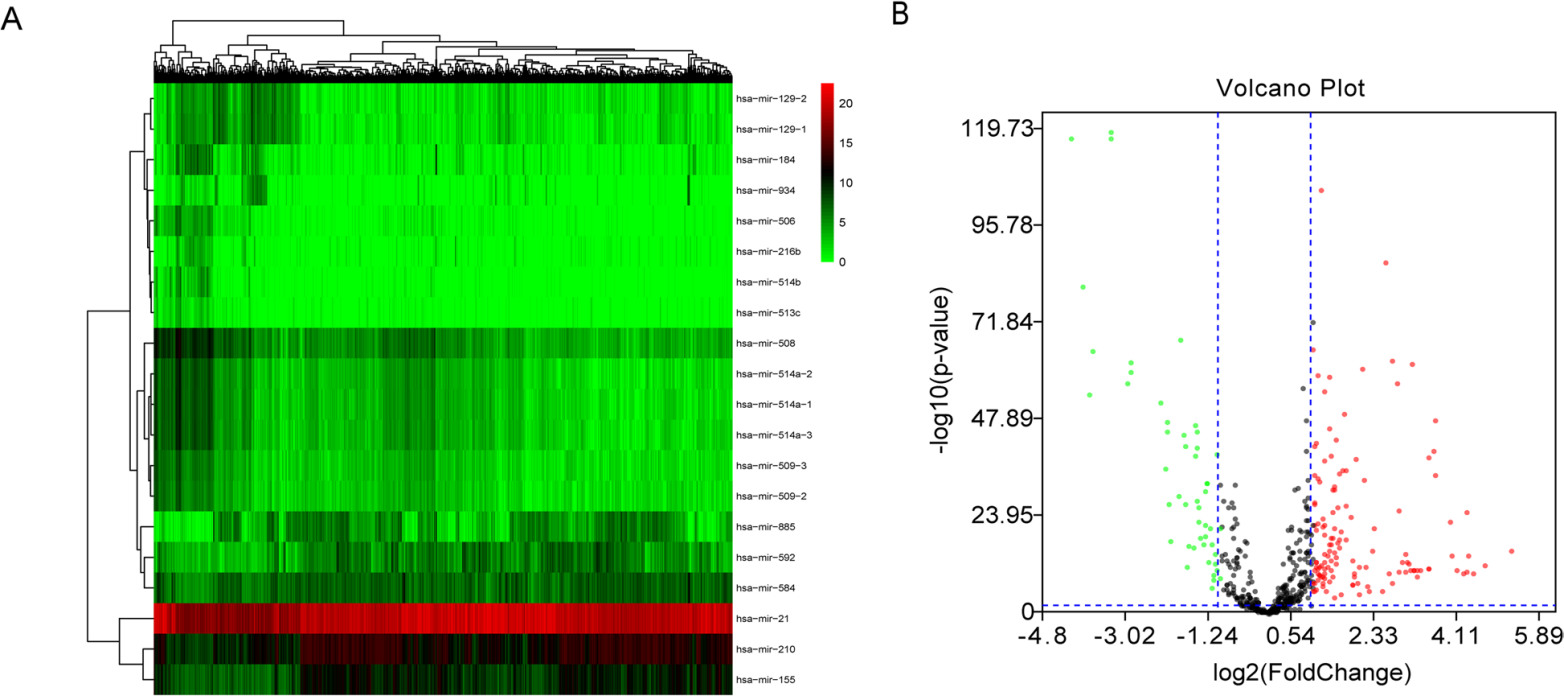
Difference analysis. **A**. The heatmap of top 20 miRNAs by difference analysis. **B**. Volcano plot of miRNAs in RCC patients. Red color indicates up-regulated expression, and green color represents down-regulated expression

Based on the TCGA database, 63 differentially expressed miRNAs (DEMs) (false discovery rate (FDR) < 0.05 and |log2fold change (log2FC) | ≥ 2) were identified using 1021 miRNAs expression profiles between 893 RCC samples and 128 adjacent normal kidney tissues (Fig. 1). These 1021 samples were matched with the clinical data downloaded from cBioPortal, and finally, 865 patients with complete clinical data were included in the study. The patients were randomly divided into a training set containing 451 samples and a validation set containing 414 samples using the caret package.

### Prognostic model construction and risk score formula

**Fig. 2:**
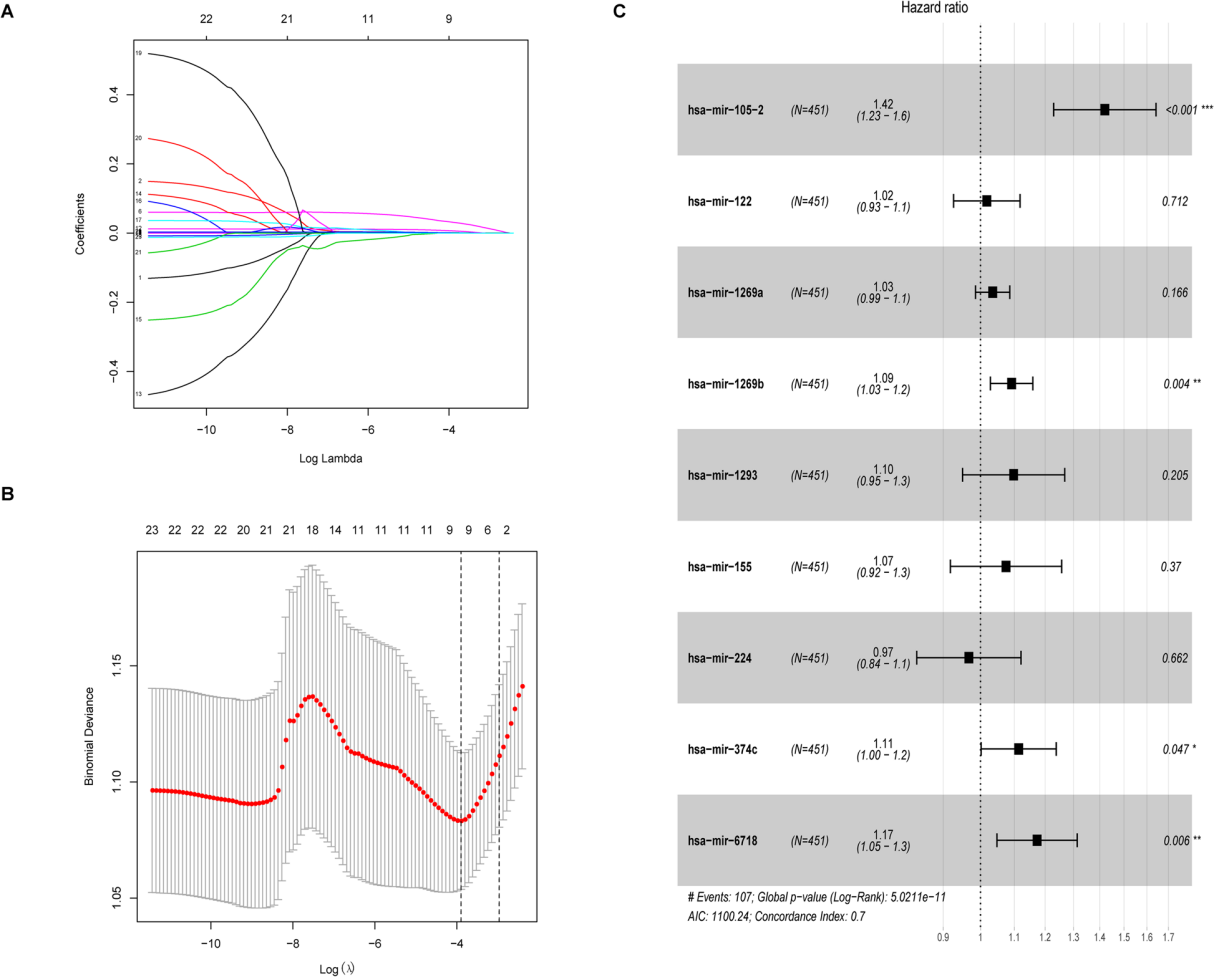
Prognostic model construction **A**. 26 cross-validations in Lambda plot. **B**. min plot. **C**. Forest plot of 9 miRNAs. ‘***’ *p* < 0.001, ‘**’ *p* < 0.01, ‘*’ *p* < 0.05

First, 26 miRNAs related to prognosis were selected through univariate cox analysis (Table S1). Based on the expression of these 26 overlapped miRNAs in the training set, the lasso analysis identified a 9-miRNA signature that was significantly associated with survival rate based on the optimal lambda value (Fig. 2). The Concordance index of the model was 0.702, and the 9 miRNAs were hsa-miR-105-2, hsa-miR-122, hsa-miR-1269a, hsa-miR-1269b, hsa-miR-1293, hsa-miR-155, hsa-miR-224, hsa-miR-374c and hsa-miR-6718. These nine miRNAs were all up-regulated expressed in RCC. Hsa-miR-105-2 was the most statistically significant with *p* < 0.001(Table 1). To facilitate the utility of the identified prognostic miRNAs in routine clinical practice, the following formula was developed to generate risk score for each patient: Risk score = 1.42033 × exprhsa-miR-105-2 + 1.01781 × exprhsa-miR-122 + 1.03488 × exprhsa-miR-1269a + 1.09167 × exprhsa-miR-1269b + 1.09789 × exprhsa-miR-1293 + 1.07460 × exprhsa-miR-155 + 0.96770 × exprhsa-miR-224+ 1.11368 × exprhsa-miR-374c + 1.17308 × exprhsa-miR-6718. Therefore, patients were divided into low-risk and high-risk groups by the same median risk score as the cut-off points in the two independent cohorts.

**Table 1:** Nine miRNAs in the prognostic model

Name	Kaplan-Meier analysis *p*-Value	Multivariate analysis			
		Coefficient	HR (95%CI)	*p*-value	
hsa-mir-105-2	0.0003	1.42033	4.756(1.229-1.641)	1.97e-06	***
hsa-mir-122	0.0117	1.01781	0.369(0.927-1.118)	0.71241	
hsa-mir-1269a	<0.0001	1.03488	1.386(0.986-1.086)	0.16589	
hsa-mir-1269b	0.0006	1.09167	2.863(1.028-1.159)	0.00420	**
hsa-mir-1293	0.0008	1.09789	1.269(0.950-1.268)	0.20460	
hsa-mir-155	<0.0001	1.07460	0.896(0.918-1.258)	0.37022	
hsa-mir-224	<0.0001	0.96770	-0.437(0.835-1.121)	0.66204	
hsa-mir-374c	0.0400	1.11368	1.990(1.002-1.238)	0.04659	*
hsa-mir-6718	<0.0018	1.17308	2.761(1.047-1.314)	0.00575	**

*HR* Hazard ratio, ‘***’ *p*<0.001, ‘**’ *p*<0.01, ‘*’ *p*<0.05

### Survival analysis

**Fig. 3:**
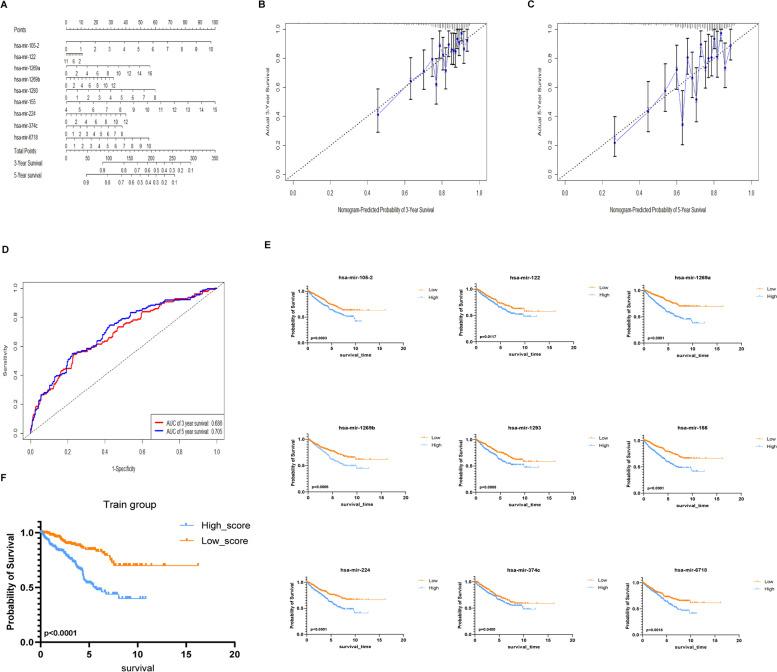
Survival analysis related to prognostic model. **A**. Nine-miRNA-based prognostic model to predict 3 and 5-year overall survival (OS) in RCC patients. **B**-**C**. Calibration plots of the nine-miRNA-based prognostic model of 3 and 5-year OS. **D**. ROC curve of 3 and 5-year OS. **E**. Impact of nine miRNAs on OS in RCC based on KM analysis. **F**. Impact of a nine-miRNA-based prognostic model in training set on OS in RCC based on KM analysis

Based on KM survival analysis, the high-risk group has a significantly worse prognosis (Fig. 3F). To further analyze the effect of the prognostic model, a 3-year and 5-year nomogram was drawn (Fig. 3A). In addition, calibration plots of the prognostic model fitted well in the training set, which indicated good calibration ability (Fig. 3B-C). The area under the ROC curve for 3-year survival in the prognostic model was 0.686. The area under the 5-year ROC curve was 0.705, which indicated that the prognostic model with low to moderate diagnostic ability (Fig. 3D). At the same time, we also conducted a separate survival analysis for these nine miRNAs. The results were that the up-regulated expressed miRNAs have a poor prognosis for RCC (Fig. 3E).

### Correlation between prognostic index and clinicopathological factors

**Fig. 4:**
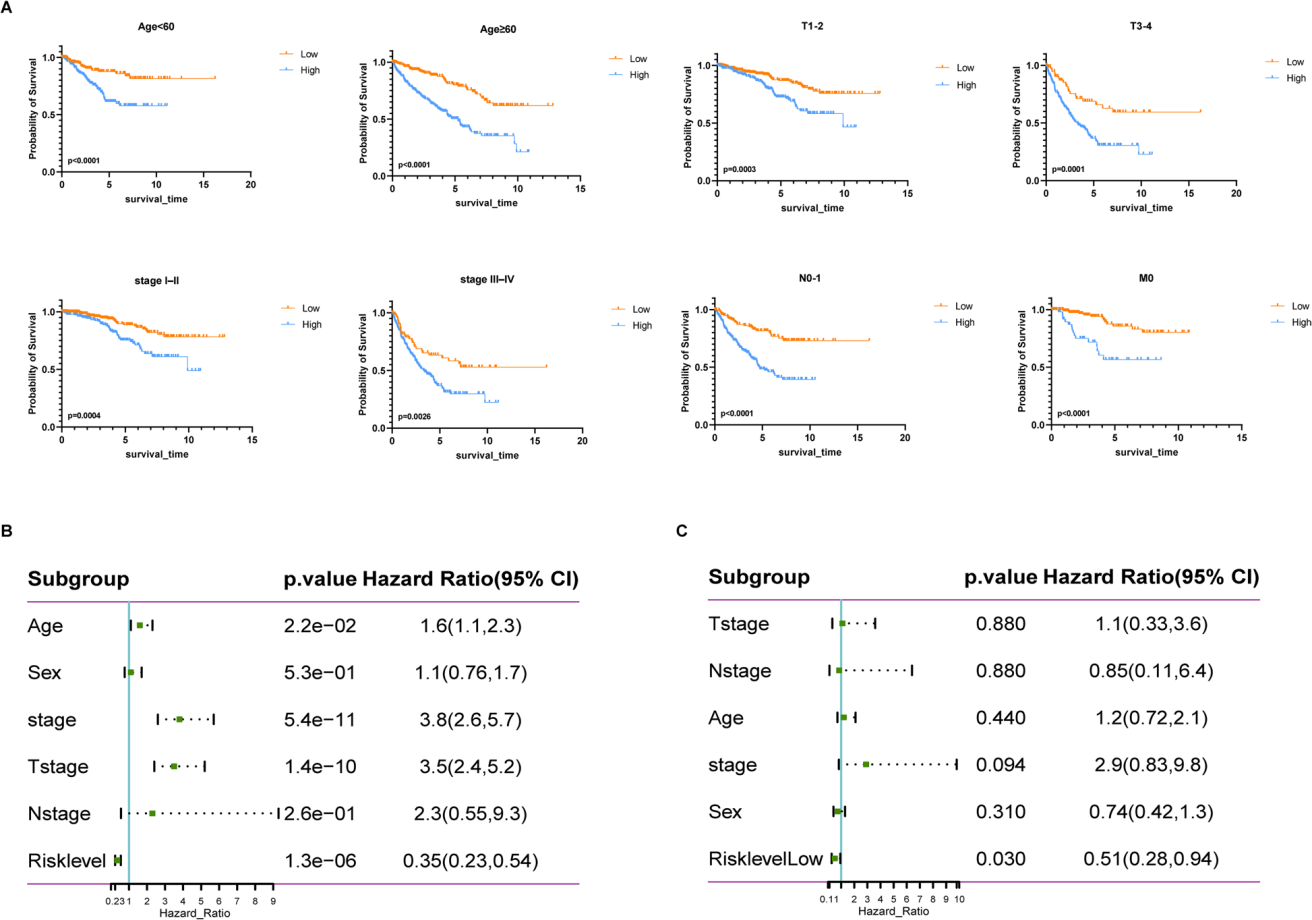
Correlation with clinicopathological factors. **A**. Impact of clinicopathological factors on OS in RCC based on KM analysis. **B**. Univariate analysis of RS and clinicopathological factors. **C**. Multivariate analysis of RS and clinicopathological factors

The clinical data of 451 patients were presented in Table 2, and based on KM survival analysis, the relationship between clinicopathological factors and OS was drawn (Fig. 4A). Clinicopathological factors had an impact on the prognosis, except for gender. Univariate and multivariate analysis further proved that our index could be used as an independent prognostic index and had a higher predictive value than the traditional tumor pathological stage (*p* < 0.05, Fig. 4B-C). (To ensure the accuracy of the results, Mstage was not included in the Cox regression analysis as lacking too much data.)

**Table 2:** Clinicopathological characteristics of RCC patients in training set

Characteristics (451)	Number (high/low)	Percentage (%)	*p*-Value (Log-rank (Mantel-Cox) test)
Age (years)			
< 60	112/103	47.67%	<0.0001
≥ 60	113/123	52.33%	<0.0001
Pathological stage			
I–II	122/171	64.97%	0.0004
III–IV	98/39	30.38%	0.0026
NA	5/16	4.65%	
T stage			
1-2	130/185	69.84%	0.0003
3-4	94/40	29.71%	0.0001
TX	0/2	4.5%	
N stage			
0-1	114/77	42.35%	<0.0001
2	2/1	0.67%	
NX	109/147	57.76%	
NA	0/1	0.22%	
M stage			
0	32/97	28.6%	<0.0001
1	4/0	0.89%	
MX	17/31	10.64%	
NA	172/98	59.87%	
Age			
Male	155/152	67.07%	ns
Female	71/73	31.93%	ns

### miRNA-mRNA regulatory network

**Fig. 5:**
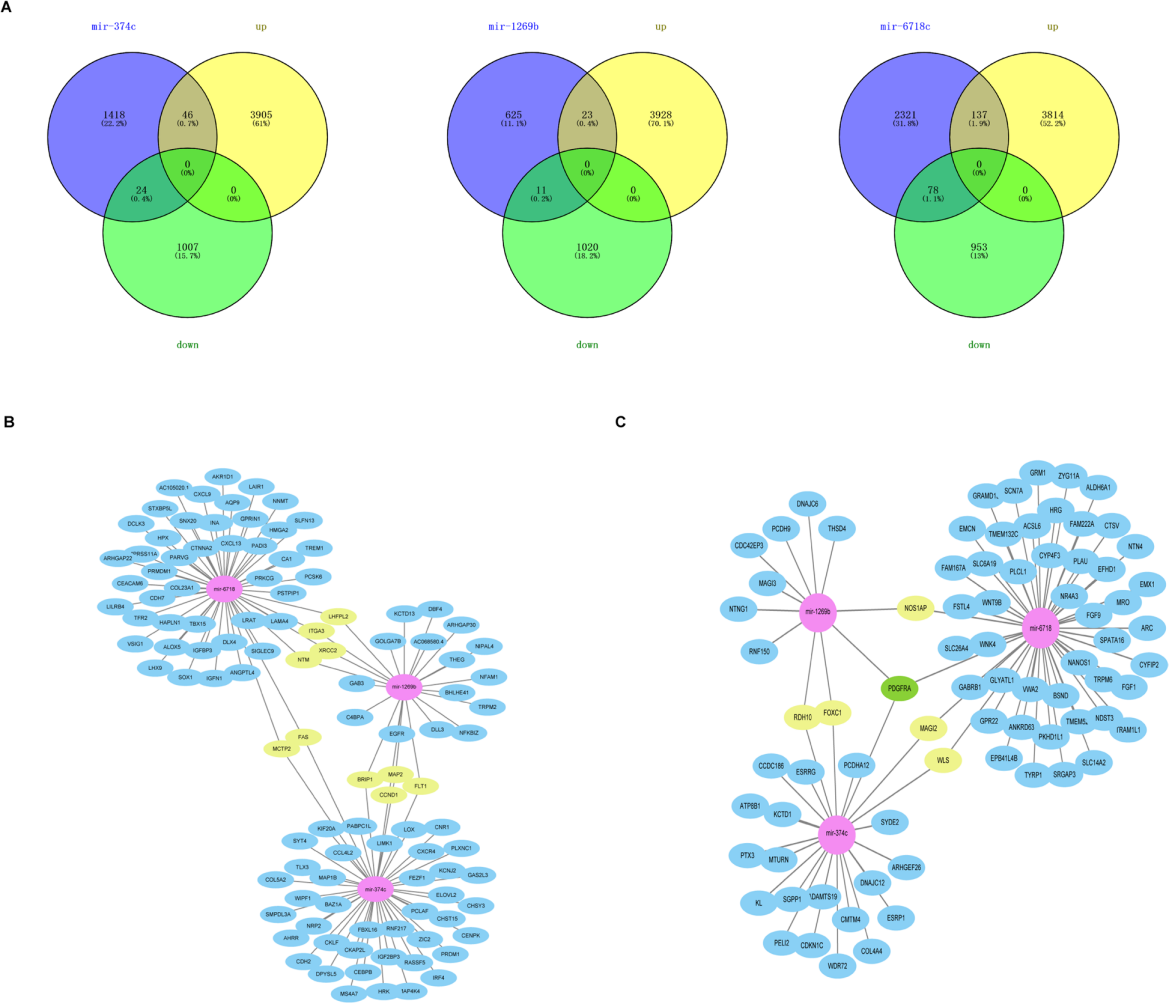
miRNA-mRNA network. **A**. The intersection of the hub gene predicted by the ENCORI website and the difference analysis result of RCC patients. **B**-**C**. The network between miRNA and the up-regulated / down-regulated genes in the intersection

Four miRNAs were selected after multivariate analysis. First, we found 1488 target genes for hsa-miR-374c, 659 target genes for hsa-miR-1269b, 2536 target genes for hsa-miR-6718, and no target genes for hsa-mirR-105-2 from ENCORI. Then, venny2.1 was used to obtain the intersection between the target gene of miRNA and the up-regulated and down-regulated genes in RCC (Fig. 5A). Finally, Cytoscape was used to make the miRNA-mRNA regulatory network, and the top 50 hub genes were drawn on the network (Fig. 5B-C). PDGFRA was the common hub gene between the three miRNAs and down-regulated genes.

### Relationship between prognostic index and tumor microenvironment (TME)

**Fig. 6:**
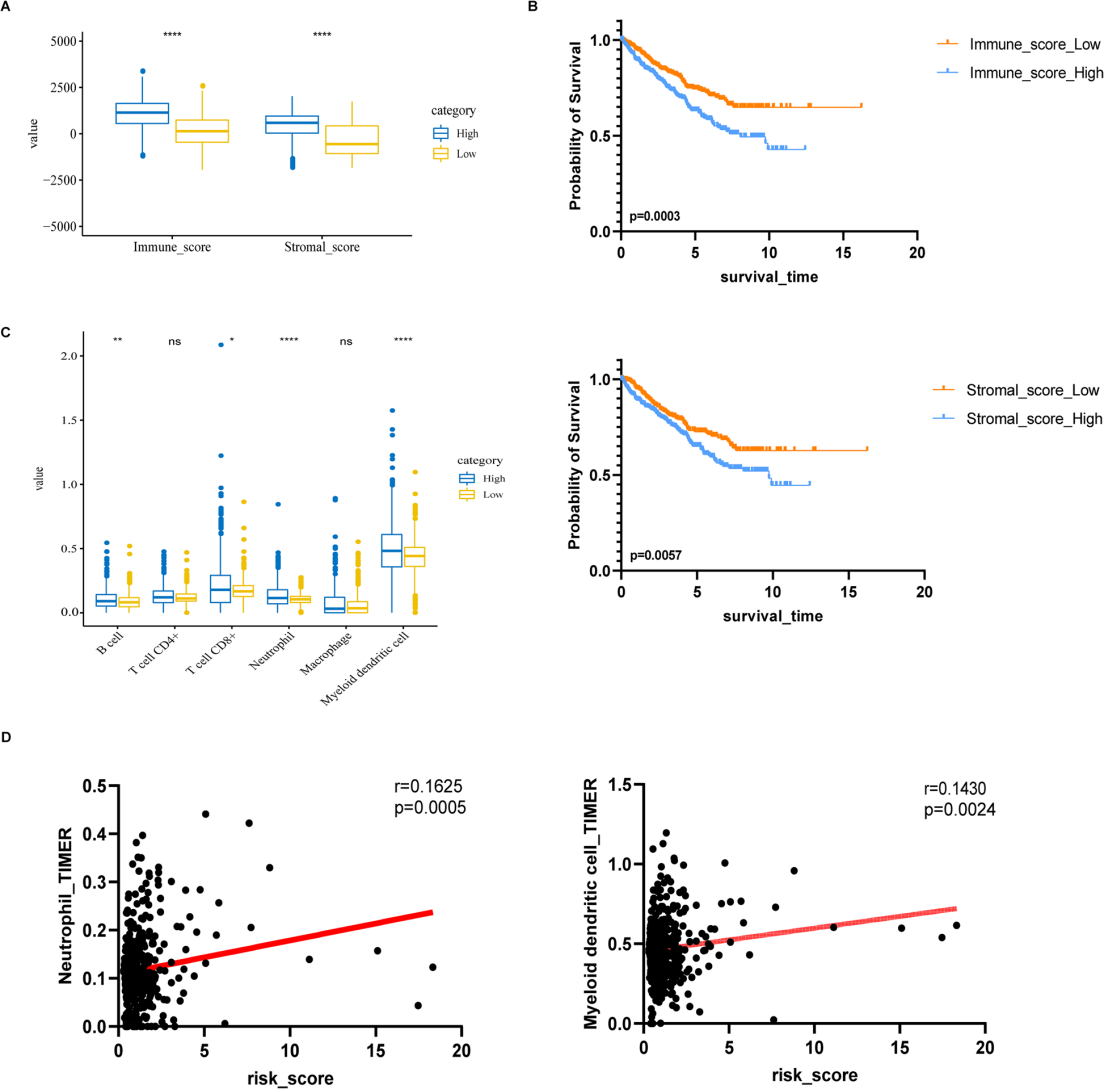
Correlation between immune infiltration and risk score. **A**. The relationship between RS and immune/stromal score. **B**. Impact of immune/ stromal score on OS in RCC based on KM analysis. **C**. The relationship between RS and immune infiltration cells. **D**. Correlation between RS and Neutrophil/ Myeloid dendritic cell

We can find that the immune score was related to survival. The median survival time of the high immune/stromal score group was shorter than that of the low immune score group (Fig. 6B). And the immune/stromal score in the high-risk group was higher than that of the low-risk group (Fig. 6A). The box plot shows that the content of B cells, T cells, CD8 +, neutrophils, and myeloid dendritic cells are significantly positively correlated with RS (*p* < 0.05, Fig. 6C). Neutrophils and myeloid dendritic cells have the strongest correlation (Fig. 6D).

### Association between prognostic index and immune checkpoint gene

**Fig. 7:**
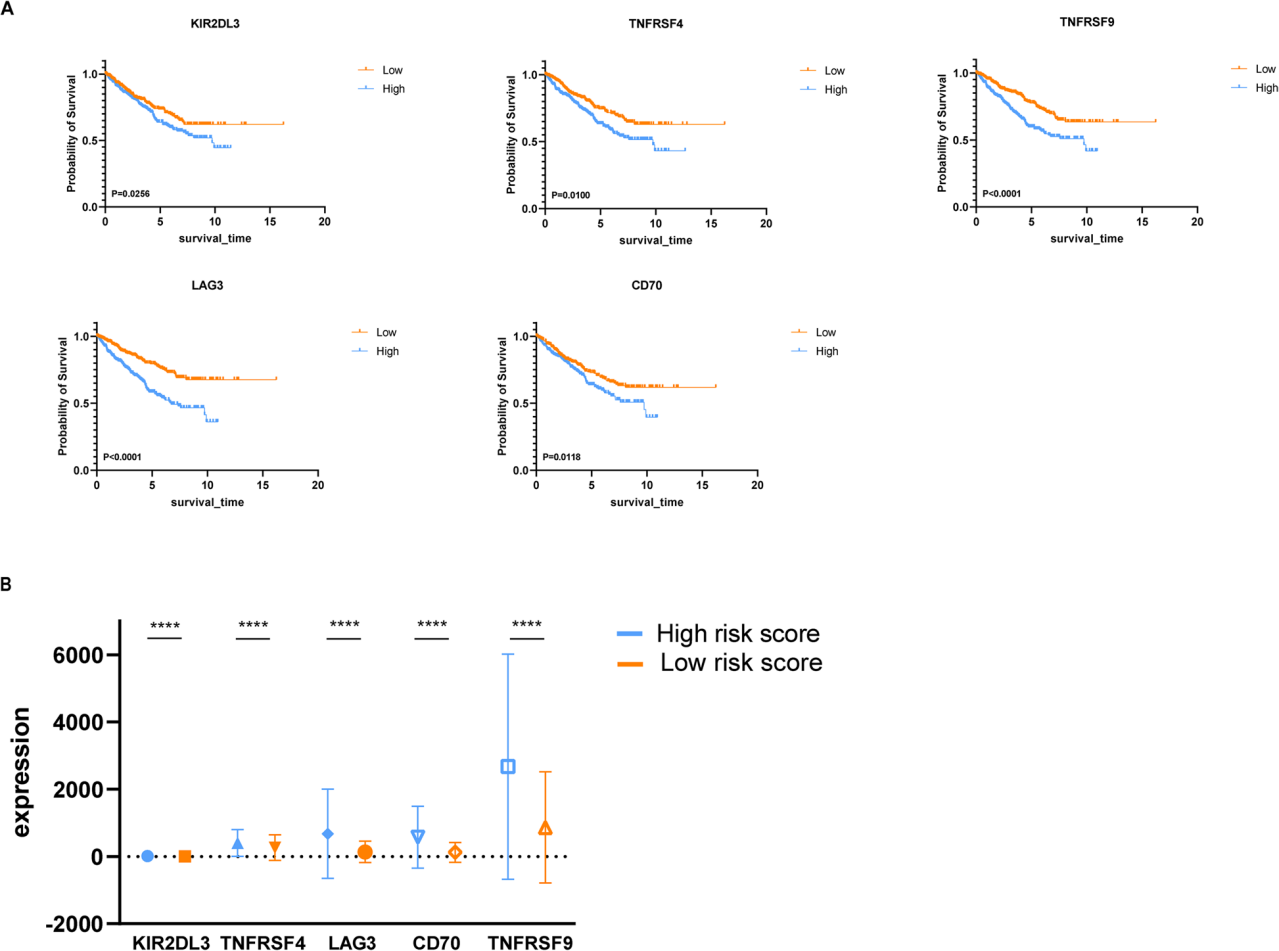
Correlation between Immune checkpoint genes and risk score. **A**. Impact of five immune checkpoint genes on OS in RCC based on KM analysis. **B**. The relationship between RS and Immune checkpoint genes

After taking the intersection between 76 ICGs and the mRNA of RCC patients, The Edger package was used for differential analysis. A total of six ICGs were screened out, KIR2DL3, TNFRSF4, LAG3, CD70 and TNFRSF9 were up-regulated genes, while ICOSLG was a down-regulated gene. Based on KM survival analysis, five up-regulated genes were found to be related to the OS, and up-regulation of gene expression indicated poor prognosis (Fig. 7A). In addition, the high-risk group of ICGs expression was higher than that of the low-risk group, which was consistent with the results of the prognosis model (Fig. 7B).

### Validation of the miRNA signature by the validation set

**Fig. 8:**
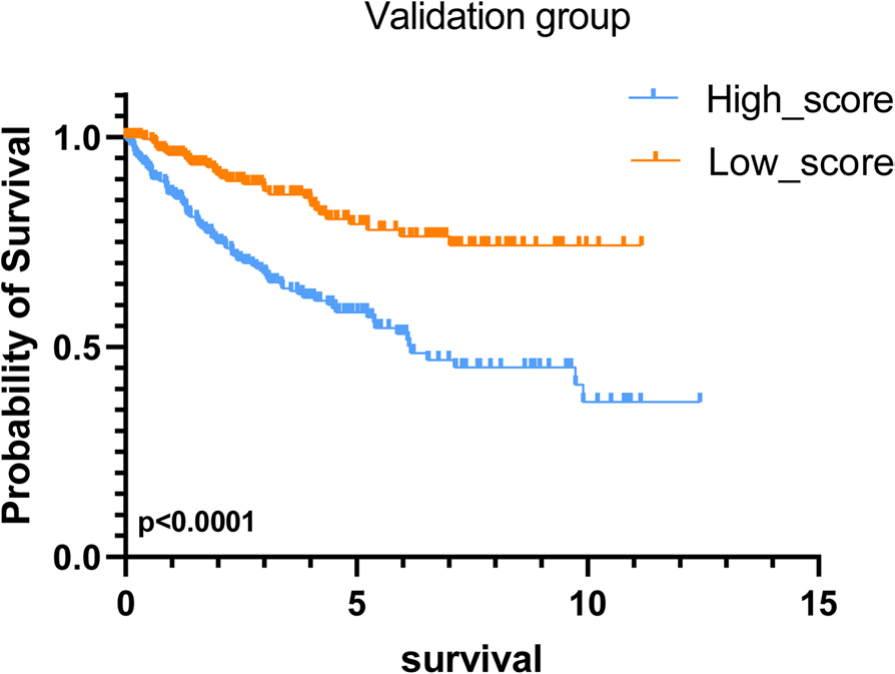
Impact of a nine-miRNA-based prognostic model in the validation set on OS in RCC based on KM analysis

To further test the ability of the prognostic model, the data in the validation set was calculated using the risk score formula and divided into high and low score groups based on the median. Based on Kaplan-Meier survival analysis, the high-risk group has a significantly worse prognosis (Fig. 8). The results were consistent with the training set, confirming the accuracy of the prognostic model.

## Discussion

There is an urgent need for a molecular marker-based method to accurately predict RCC patients’ survival rates. The major established risk factors for RCC based on clinical factors include hypertension, excess body weight, and smoking [19]. Increasing evidence shows that miRNAs play a vital role in RCC prognosis. In this study, we established a prognostic model of RCC containing nine miRNAs, and all these miRNAs have been shown to have a harmful effect on survival time. Their high expression leads to a shorter survival time.

In one literature report, we only found one target gene of hsa-miR-105-2 as CSAG1 [20]. Chondrosarcoma-associated gene one protein (CSAG1) depletion results in delayed mitotic progression and multipolar mitotic exit. Depletion of CSAG1 disrupts centrosomes and leads to multipolar spindles, particularly in cells with compromised p53 function [21]. It has been reported that high hsa-miR-122 expression promotes the malignant phenotype of clear cell RCC (ccRCC) by targeting occludin [22]. hsa-miR-1269b can promote the proliferation and migration of hepatocellular carcinoma, drives Cisplatin Resistance of Human Non-Small Cell Lung Cancer (NSCLC) [23–25]. Few studies related to hsa-miR-1269a and cancer were found. It was reported that hsa-miR-155 and hsa-miR-224 worked as cancer suppressors in RCC [26–29]. hsa-miR-374c-5p plays a crucial role in the invasion and migration of cervical cancer by acting on the Foxc1/snail pathway [30]. hsa-miR-6718 may be related to DNMT3A-mutant acute myeloid leukemia patients [31]. Due to the inhibitory and regulatory effects of miRNAs on downstream mRNAs, in recent decades, scientists have been committed to developing robust and safe targeting methods to restore these inhibitory miRNAs in cancer cells [32]. miRNAs play the role of a sponge to silence mRNA. Perhaps these miRNAs can be used as therapeutic targets to inhibit tumor growth.

Based on KM survival analysis, it can be found that the model can predict the prognosis of RCC. The survival time of the high-risk group was much shorter than that of the low-risk group. Moreover, the high expression of the nine miRNAs in the model all predicts a poor prognosis. The area under the ROC curve suggests that the model with moderate diagnostic ability. Clinicopathological factors had an impact on the prognosis, except for gender. Univariate and multivariate analysis further proved that our index could be an independent prognostic index and had a higher predictive value than the traditional tumor pathological stage. Moreover, the three-year and five-year calibration curves had good accuracy and consistency, which all prove that the prediction performance of this model was better. Based on the results of multivariate analysis, we expected the target genes of 4 miRNAs with *p* < 0.05 and found that PDGFRA was the common down-regulated gene of hsa-miR-374c, hsa-miR-6718, and hsa-miR-1269b. This gene may be used as a breakthrough point in the mechanism of miRNA-mRNA for RCC, and improve the prognosis of patients by inhibiting upstream miRNAs. Platelet-derived growth factor receptor alpha (PDGFRA) encodes a cell surface tyrosine kinase receptor for the platelet-derived growth factor family members. Studies suggest that this gene plays a role in organ development, wound healing, and tumor progression, whereas mutant-PDGFRA is potently oncogenic [33, 34].

RCC can be treated with ablation (destruction of the malignant tissue with heat or cold), partial or radical nephrectomy (removal of the kidney), or active surveillance (monitoring of tumor growth with periodic radiographic studies) [35]. In recent years, the approval of immunotherapeutic drugs and immunotherapy-based combination strategies has revolutionized the treatment of patients with advanced renal cell carcinoma (aRCC) [36]. In order to study the impact of TME and immune infiltration on RCC, we conducted KM analysis and correlation analysis. It was found that the group with a high immune/stromal score indicated a poor prognosis, which was positively correlated with RS. Furthermore, Infiltration levels of B cell, T cell CD8 +, Neutrophil, and Myeloid dendritic cell were higher in a high-risk group and were significantly positively correlated with RS. Neutrophils are recruited into the tumor microenvironment by the chemokine receptor system and play a role in tumor progression and metastasis [37]. The cytokines and chemokines secreted by the TME and tumor-associated immune cells promote a sustained and non-resolving tumor-associated inflammation. In addition, regulatory B cells increase tumor activity by regulating immune cells [38]. These may be the reason why the increase of these cells was negatively correlated with the prognosis. It was consistent with previous studies on immune infiltration.

ICGs play a key role in evading self-reactions and represent a new goal for t developing of cancer treatments. For example, Nivolumab, a programmed death 1 (PD-1) immune checkpoint inhibitor monoclonal antibody, was approved as a monotherapy for aRCC after treatment with a VEGF-targeted agent [39]. In our study, a total of 5 immune checkpoints related to RCC were found, and all of them were up-regulated genes. Among them, LAG3 and TNFRSF9 have the strongest correlation with prognosis. Lymphocyte activating 3 (LAG3) belongs to the Ig superfamily and contains four extracellular Ig-like domains and represents an inhibitory receptor, mainly found on activated immune cells and involved in the exhaustion of T cells in malignant diseases. In cancer, T cells are continuously exposed to antigens, leading to the gradual loss of cytokine production and the ability of CD8+ T cells to specifically kill tumor cells [40]. LAG3 is expressed on regulatory T cells in tumors and induces the production of IL-10 and TGF-β1, which helps tumor immune escape. Therefore, blocking LAG3 on regulatory T cells may reduce their inhibitory function and lead to a revival of CD8 + tumor-infiltrating lymphocytes activity [41]. Therefore, LAG3 is suitable as a clinical target to block cancer progression [42]. In our study, the high expression of LAG3 indicates a poor prognosis, and it is positively correlated with RS, which is consistent with these studies. TNF receptor superfamily member 9(TNFRSF9) plays a vital role in regulating the function of CD8^+^ T cells. It has been reported that TNFRSF9+ CD8+ T cells have exhaustion and effector phenotypes, identified as poor prognostic factors for ccRCC [43]. In our study, TNFRSF9 and T CD8+ cell scores were higher in the high-risk group than in the low-risk group. This may be the reason for the poor prognosis of the high-risk group. The influence of CD70 molecule (CD70), TNF receptor superfamily member 4 (TNFRSF4) and killer cell immunoglobulin like receptor, two Ig domains and long cytoplasmic tail 3 (KIR2DL3) on the prognosis of RCC is not so obvious as the first two genes. In most studies, these three genes need to work together with other genes, and they need to be verified as immunotherapy targets.

In the study, we constructed a nine-miRNAs prognostic model, which has been successfully verified in the validation data set and shows reliable predictive performance and significant clinical value in a comprehensive analysis with clinicopathological factors. We found PDGFRA, a common target gene of hsa-miR-6718, hsa-miR-1269b and hsa-miR-374c, and five genes related to ICGs.

In conclusion, we successfully constructed a nine-miRNAs prognostic model with good predictive power. Moreover, this model was closely related to the TME, immune infiltration and ICGs of RCC, and it is hoped that it can provide new ideas for RCC immunotherapy.

## Supplementary Information

**Additional file 1.**

## Data Availability

The data that support the findings of this study are available in The Cancer Genome Atlas (TCGA) and Gene Expression Omnibus (GEO).

## Abbreviations

RCC: Renal cell carcinoma
TCGA: The cancer genome atlas
TME: Tumor microenvironment
KM: Kaplan–Meier
RS: Risk score
OS: Overall survival
HR: Hazard ratio
ICGs: Immune checkpoint genes
