# Supplementary material for: A novel nine-microRNA-based model to improve prognosis prediction of renal cell carcinoma
Source: BMC Cancer. 2022 Mar 12;22:264. doi: 10.1186/s12885-022-09322-9 (PMC8918330; doi:10.1186/s12885-022-09322-9)
Supplement: Supplementary file 1 — Additional file 1. [file 12885_2022_9322_MOESM1_ESM.docx]

**Supplemental Digital Content -Table1.** The result of univariate cox analysis

| **miRNA name** | **HR** | **P value** |
| --- | --- | --- |
| \| hsa-mir-21 \| \| --- \| \| hsa-mir-216b \| \| hsa-mir-210 \| \| hsa-mir-155 \| \| hsa-mir-122 \| \| hsa-mir-1293 \| \| hsa-mir-224 \| \| hsa-mir-6718 \| \| hsa-mir-518c \| \| hsa-mir-526b \| \| hsa-mir-374c \| \| hsa-mir-525 \| \| hsa-mir-519a-2 \| \| hsa-mir-512-2 \| \| hsa-mir-519a-1 \| \| hsa-mir-518b \| \| hsa-mir-520b \| \| hsa-mir-512-1 \| \| hsa-mir-767 \| \| hsa-mir-1269a \| \| hsa-mir-105-2 \| \| hsa-mir-1269b \| \| hsa-mir-105-1 \| | \| 1.216013781 \| \| --- \| \| 1.216622601 \| \| 1.160987623 \| \| 1.298206569 \| \| 1.067156424 \| \| 1.333543783 \| \| 1.260978841 \| \| 1.165702495 \| \| 1.19284301 \| \| 1.128059674 \| \| 1.125363201 \| \| 1.276559753 \| \| 1.307805577 \| \| 1.289794917 \| \| 1.294111517 \| \| 1.187573348 \| \| 1.331215548 \| \| 1.235079349 \| \| 1.231324893 \| \| 1.109310449 \| \| 1.257998408 \| \| 1.086132861 \| \| 1.246784468 \| | \| 0.000359984 \| \| --- \| \| 0.037670083 \| \| 0.000663941 \| \| 2.67E-10 \| \| 0.00605115 \| \| 6.93E-10 \| \| 5.59E-08 \| \| 8.23E-06 \| \| 0.009537242 \| \| 0.040465492 \| \| 0.000282704 \| \| 0.000589247 \| \| 3.12E-05 \| \| 0.000184648 \| \| 1.59E-06 \| \| 0.036922856 \| \| 1.66E-05 \| \| 0.007285108 \| \| 3.35E-07 \| \| 2.55E-12 \| \| 1.38E-08 \| \| 9.47E-05 \| \| 8.17E-08 \| |

*miRNA, microRNA; HR, hazard ratio*
